# The Proportion of Regulatory T Cells in Patients with Ankylosing Spondylitis: A Meta-Analysis

**DOI:** 10.1155/2019/1058738

**Published:** 2019-10-23

**Authors:** Na-Lin Lai, Sheng-Xiao Zhang, Jia Wang, Jia-Qian Zhang, Cai-Hong Wang, Chong Gao, Xiao-Feng Li

**Affiliations:** ^1^Department of Rheumatology, The Second Hospital of Shanxi Medical University, 382 Wuyi Road, Taiyuan, Shanxi 030001, China; ^2^Department of Pathology, Brigham and Women's Hospital, Harvard Medical School, Boston, MA, USA

## Abstract

**Objective:**

Accumulating evidence indicates that regulatory T cells (Tregs) may be involved in the pathogenesis of ankylosing spondylitis (AS). As different markers have been used to identify Tregs, some studies on the proportions of Tregs in AS patients have generated considerable controversy. To clarify the status of Tregs in such patients, we determine the proportion changes of peripheral Tregs during development of the disease, with different cellular markers.

**Methods:**

We systematically searched Embase, PubMed, Cochrane, Web of Knowledge, FDA.gov, and Clinical Trials.gov for the studies reporting the proportion of Tregs in AS patients. Using the PRISMA guidelines, we performed a random-effects meta-analysis of the frequencies of peripheral Tregs defined in different ways. Inconsistency was evaluated using the *I*-squared index (*I*^2^), and publication bias was assessed by examining funnel plot asymmetry using the Begger and Egger tests.

**Results:**

A total 29 studies involving 1732 participants were included in the meta-analysis. Their conclusions of using the diversity of Tregs surface markers were inconsistent with each other. No significant difference in the proportions of Tregs was evident regardless of the definitions used [−0.709, (−1.455, 0.037, *p* = 0.063), *I*^2^ = 97.3%]. Six studies used “single CD25-positive” cells as Tregs, which revealed a significant increase in AS patients compared with healthy blood donors [0.736, (0.138, 1.334), *p* = 0.016, *I*^2^ = 80.7%]. Notably, the proportions of “CD4^+^CD25^+^FOXP3^+^,” “CD4^+^CD25^high^CD127^low/−^,” or “CD4^+^CD25^+^CD127^low^” T cells were lower in AS patients [−2.856, (−4.645, −1.066), *p* = 0.002; −1.812, (−2.648, −0.977), *p* < 0.001; −1.12, (−1.605, −0.635), *p* < 0.001]. Tregs defined as “CD25^high^,” “CD25^bright^,” “CD25^bright/high^CD127^low/−^,” “CD4^+^FOXP3^+^,” “CD4^+^CD25^high^FOXP3^+^,” and “CD4^+^CD25^+^CD127^−^” did not differ in proportion between AS patients and healthy blood donors.

**Conclusions:**

The levels of Tregs varied based on the cellular identification markers used. The proportions of CD4^+^CD25^+^FOXP3^+^Tregs, CD4^+^CD25^high^CD127^low/−^, or CD4^+^CD25^+^CD127^low^ in blood of AS patients were significantly decreased as compared with those in healthy blood donors, and our findings lend support to the idea that the Treg status of AS patients is important. And we recommend the above as the best definition of Tregs when evaluating the status of such patients.

## 1. Introduction

Ankylosing spondylitis (AS) is a common inflammatory rheumatic disease that affects the axial skeleton, causing characteristic inflammatory back pain, asymmetrical peripheral oligoarthritis, enthesitis, and specific organ involvement such as anterior uveitis, psoriasis, and chronic inflammatory bowel disease, which can lead to structural and functional impairments and a decrease in quality of life [1]. To date, the disease etiology remains unclear. Reduced proportion and deficient function of CD4^+^ regulatory T cells (Tregs, with immune modulation and suppression) have been implicated in the pathogenesis of different immune-mediated rheumatic diseases [2–4]. In the case of AS, few studies have been carried out to analyze the levels of Tregs in the peripheral blood of patients; however, low percentages [5–8] or functional impairment of Tregs [9, 10] has been reported in the peripheral blood (PB) of patients with AS, suggesting an imbalance between Tregs and the adaptive immune response. Moreover, AS patients treated with anti-TNF therapy showed similar levels of Treg cells to those observed in healthy subjects [11]. These data suggest a possible role of Tregs in AS.

However, initial studies of Treg status in PB of patients with AS are controversial. One reason for the inconsistencies may be the multiple phenotypes of Tregs, which have been identified using different markers [12]. Tregs were first described as a peripheral CD4^+^ subset expressing interleukin- (IL-) 2 receptor alpha chains (CD25) [13]. As early as 2004, Cao et al. [14] used CD4^+^CD25^bright^ to identify peripheral Tregs in peripheral blood of AS patients. However, CD25 was expressed not only on Tregs but also on activated cells lacking regulatory functions, although the CD4^+^ T cell subset expressed the highest levels of CD25 (CD4^+^, CD25^high^) and exhibited in vitro immunosuppressive features [15]. Forkhead box protein P3 (FOXP3), a transcription factor expressed at high levels in authentic Tregs, plays a key role in Treg development and is thought to be one of the most specific Treg cell markers [16]. Since 2008, scholars have been studying the proportion and function of peripheral FOXP3^+^Tregs of AS patients [9, 17]. However, the marker cannot be used to sort live cells, as the protein is intracellular. In addition, CD127, the alpha chain of the IL-7 receptor, was reported to be upregulated on human T cells after activation and downregulated on Tregs [18]. Thus, costaining for CD127 and CD25 has been proposed to efficiently discriminate between Tregs and activated T cells [19]. The study of CD4^+^CD25^+^CD127^−^Tregs in AS patients began in 2011. Zhao et al. used CD25^+^CD127^−^ to define peripheral Tregs in new-onset AS patients firstly [6]. Furthermore, CD8^+^CD122^+^ T cell is a newly discovered natural immune regulatory T cells with immune negative regulation function [20], which may be involved in the pathogenesis and disease progression of AS [21]. The available data on the proportions and phenotypes of Tregs of AS patients are contradictory; some studies using the same or different markers to analyze peripheral Tregs of AS patients have obtained different or even opposite results [22–25].

To better understand Treg malfunctions in patients with AS, we meta-analyzed reports documenting the proportion of peripheral Treg cells among CD4^+^ T cells in the PB of patients with AS, as well as healthy blood donors in this study.

## 2. Methods

### 2.1. Data Sources and Searches

This meta-analysis was consistent with that of the Preferred Reporting Items for Systematic Reviews and Meta-Analyses (PRISMA) Statement, and it had been registered at the International Prospective Register of Systematic Reviews (PROSPERO) (CRD42019120790). We searched for relevant studies published between January 1, 1950, and November 1, 2018, using PubMed, Embase, Cochrane, Web of Knowledge, Clinical Trials.gov, and FDA.gov, with no restrictions in terms of the primary outcome or publication language. We used the MeSH terms “Spondylitis, Ankylosing” and “T−lymphocytes, regulatory” and their combination. All potentially eligible studies were considered except for reviews and murine experiments. Key articles listed in the references were retrieved manually.

### 2.2. Study Selection and Data Extraction

The inclusion criteria were evaluation of the proportion of Tregs among CD4^+^ T cells of AS patients using the 1984 Modified New York AS Criteria [26], available as a full text article, and information on the number of patients and healthy blood donors. Two investigators independently selected and identified relevant publications, and a third investigator resolved any disagreements. The evidence levels of the studies were assessed based on the 2011 guidelines of the Oxford Centre for Evidence-Based Medicine. Quality assessment was done with the Newcastle-Ottawa Quality Assessment Scale, which can be used to assess the quality of nonrandomized studies.

We recorded patient baseline characteristics and their country of origin, the year of publication, the number of patients and healthy blood donors, the definition of Tregs used (including CD4^+^CD25^+^, CD4^+^CD25^bright^, CD4^+^CD25^high^, CD25^low/−^FOXP3^+^, FOXP3^+^, CD25^+^FOXP3^+^, CD25^high^FOXP3^+^, CD25^+^CD127^−^, CD25^bright/high^CD127^low/−^, and CD25^high^CD127^low/−^FOXP3^+^), and the mean (or median) and standard deviation (SD) of the proportion of Tregs among CD4^+^ T cells. Data on the proportion of Tregs in patients with HLA-B27(+) and HLA-B27(−) were also extracted.

### 2.3. Statistical Analysis

For continuous outcomes (the proportions of Tregs among CD4^+^/CD8^+^ T cells of patients with AS and healthy blood donors), we calculated standardized mean differences (SMDs) and compared these values by using a random-effects model (REM) (the DerSimonian and Laird method) [27]. When Treg percentages were reported as medians with interquartile ranges (IQRs), we calculated means and SD (SD = IQR/1.35). The Cochrane chi-squared test was used to explore between-study heterogeneity. As heterogeneity was high (*I*^2^ > 75%), we drew forest plots and performed subgroup analyses to explore the possible effects of study characteristics on outcomes. Publication bias was assessed by examining funnel plot asymmetry using the Begger and Egger tests (*p* ≥ 0.05). A preplanned sensitivity analysis was performed by omitting each study individually and calculating the remaining pooled effect. All statistical analyses were conducted using Stata software (ver. 12.0).

## 3. Results

### 3.1. Study Characteristics

**Figure 1:**
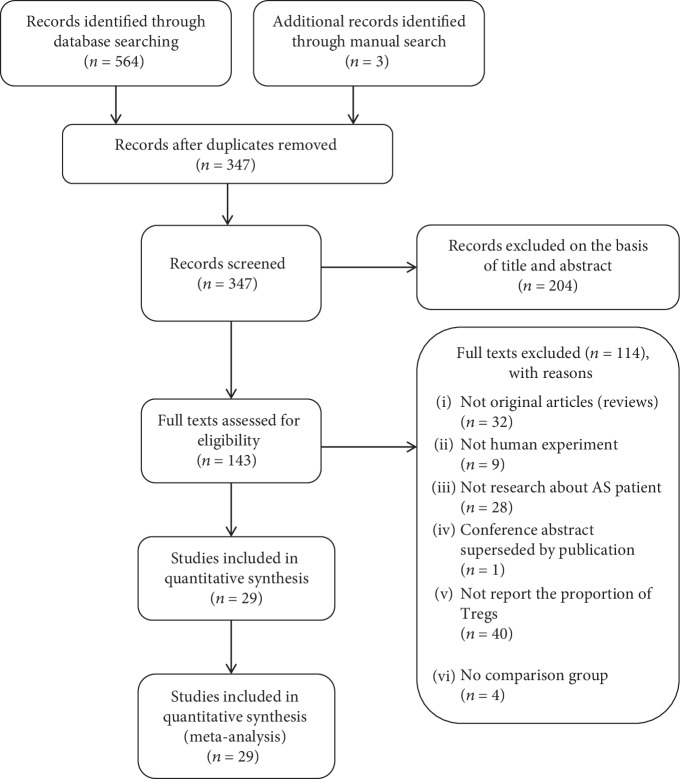
The study selection process.

We identified 564 studies. And 29 of them (with data on 980 patients and 752 healthy blood donors) were included in the analysis (Figure 1), and all of them used a reliable flow cytometric analysis to detect the proportions of peripheral Tregs. The details are shown in Table 1. The average age of the AS patients was between 24.8 and 52.13 years, the proportion of males ranged from 0 to 100%, the average disease duration was from 1.6 to 13.3 years, the average erythrocyte sedimentation rate (ESR) was from 15.2 to 57.3 mm/hour, the average C-reactive protein (CRP) was from 6.63 to 77.1 mg/l, and the Bath Ankylosing Spondylitis Disease Activity Index (BASDAI) [28] from was 1.19 to 51.94. Patients were treated with glucocorticoids, NSAIDs, DMARDs, immunosuppressants including cyclophosphamide (CTX) and cyclosporine, and biological agents. All healthy blood donors were age and sex matched, healthy, and without any autoimmune disease. All studies were poor-quality case-control studies or case series; thus, they were all of evidence level 4. We regarded all studies as case-control studies and scored them using the Newcastle-Ottawa Quality Assessment Scale (NOQAS); all studies had a score of 3–5.

### 3.2. The Proportion of Peripheral Tregs of AS Patients

**Figure 2:**
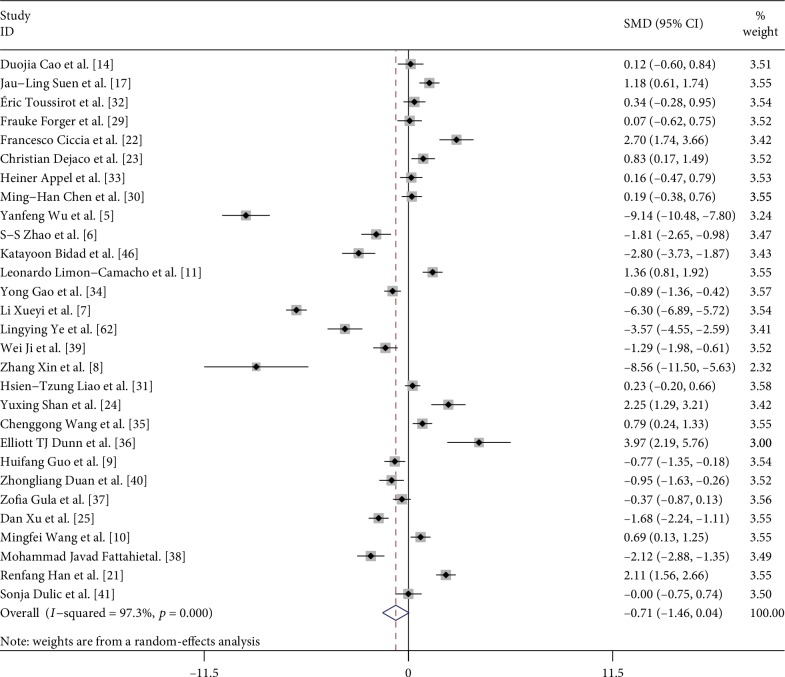
Forest plot of the overall meta-analysis of regulatory T cell (Treg) proportions in peripheral blood (PB), regardless of the Treg definitions used, between ankylosing spondylitis (AS) patients and healthy blood donors (HD).

First, we performed a meta-analysis of the proportion of Tregs between AS patients and control subjects in all studies, neglecting the definition methods of Tregs (Figure 2). Unexpectedly, there was no significant difference in the proportion of Tregs in PB between AS patients and healthy blood donors in all studies [−0.709, (−1.455, 0.037, *p* = 0.063)]. Besides, there was statistically significant heterogeneity between studies (*I*^2^ = 97.3%). In this analysis, there was no publication bias on Egger test (*p* = 0.227).

We hypothesized that the cause of unexpected results may be the different definition methods of Tregs. Thus, we performed a subgroup analysis based on the Treg definitions to explore the potential sources of heterogeneity. First, we analyzed studies that identified Tregs only as “CD25-positive” in CD4^+^ T cell subpopulations. A pooled analysis of all 6 trials [14, 22, 23, 29–31] revealed a significant increase in the proportion of Tregs in AS patients compared with healthy blood donors [0.736, (0.138, 1.334), *p* = 0.016] with statistically significant between-study heterogeneity (*I*^2^ = 80.7%, *p* < 0.001) and no significant between-study publication bias detected by the Egger test (*t* = 0.72, *p* = 0.513). In detail, we found a significant increase in the proportion of Tregs between AS patients and healthy blood donors when Tregs were defined as “CD4^+^CD25^+^” cells [0.846, (0.401, 1.291), *p* < 0.001] [31]. However, the proportion of Tregs defined as “CD4^+^CD25^high^” cells [0.892, (−0.078, 1.862), *p* = 0.071] [22, 23, 29, 30] and as “CD4^+^CD25^bright^” cells [0.123, (−0.596, 0.842), *p* = 0.737] [14] did not differ significantly between patients and healthy blood donors (Table 2).

Then, we analyzed studies in which Tregs were defined as “FOXP3^+^” cells. A pooled analysis of all 18 trials [5, 7, 8, 11, 17, 25, 31–38] revealed a significant decrease in the proportion of such Tregs between AS patients and healthy blood donors [−1.004, (−1.966, −0.042), *p* = 0.041]. Statistically significant heterogeneity was evident among the studies (*I*^2^ = 97.9%, *p* < 0.001). The Egger test detected no publication bias (*t* = 0.97, *p* = 0.795). Among the studies, 9 [5, 7, 8, 25, 31, 32, 34, 35, 38] used “CD4^+^CD25^+^FOXP3^+^” to define Tregs, which showed that the proportion of Tregs in AS patients appeared to be lower than in healthy blood donors [−2.856, (−4.645, −1.066), *p* = 0.002]. However, pooling of these data with those of other studies [17, 31] identifying Tregs as “CD4^+^CD25^low/−^FOPX3^+^” cells revealed a higher proportion of Tregs in patients than in healthy blood donors [0.683, (0.161, 1.206), *p* = 0.01]. Tregs were identified as simply “FOXP3^+^” cells [11, 33, 37]; and “CD25^high^FOXP3^+^” cells [9, 17, 36] [0.383, (−0.663, 1.429), *p* = 0.473; 0.868, (−0.756, 2.492), *p* = 0.295] were not shown to be significantly different between patients and healthy blood donors (Table 2).

Finally, the other four groups [6, 39–41] that used “CD127-negative” in CD4^+^ T cell subgroups to define Tregs showed that such cell numbers decreased in AS patients [−1.003, (−1.713, −0.294), *p* = 0.006] with statistical heterogeneity (*I*^2^ = 73.1%, *p* = 0.011) and no publication bias (*t* = −0.37, *p* = 0.747). More specifically, pooling the data of studies in which Tregs were identified as “CD4^+^CD25^high^CD127^low/−^” cells [6] and “CD4^+^CD25^+^CD127^low^” cells [39, 40] revealed a significant decrease between AS patients and healthy blood donors [−1.812, (−2.648, −0.977), *p* < 0.001; −1.12, (−1.605, −0.635), *p* < 0.001], but no significant difference was observed when Tregs were defined as “CD4^+^CD25^+^CD127^−^” cells [−0.004, (−0.751, 0.744), *p* = 0.992] [41] (Table 2).

Due to the heterogeneity in the meta-analysis, the random-effects model was applied in preparing forest plots. We hypothesized that the significant heterogeneity might have been caused by differences in the experimental methods, and clinical type and severity of disease among the different studies.

### 3.3. Disease Activity and the Proportion of Tregs in PB

**Figure 3:**
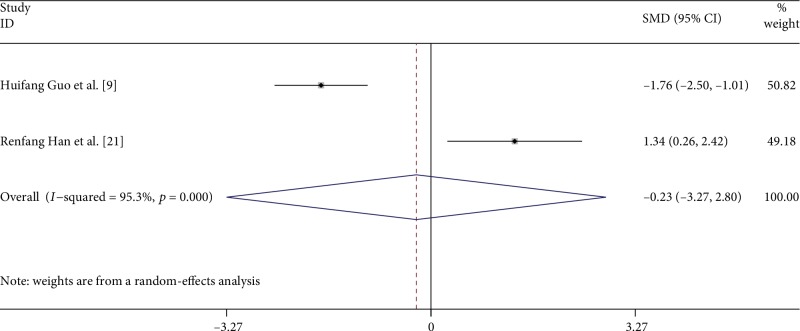
Forest plot of the overall meta-analysis of regulatory T cell (Treg) proportions in peripheral blood (PB), regardless of the Treg definition used, in patients with active and stable AS.

To further assess the effect of disease activity, we analyzed 2 studies [9, 21] that reported the proportion of Tregs in active and stable AS patients regardless of the Tregs definitions used (Figure 3). All of these 2 studies used the Ankylosing Spondylitis Disease Activity Score (ASDAS) [42–44] to evaluate the disease activity. Guo H. et al. [9] found no significant differences in the percentages of Tregs among patients with active AS and patients with stable AS, but Han R. et al. [21] showed a significant increase. We found no difference in the proportion of Tregs in patients with active compared with stable AS [−0.234, (−3.267, 2.799), *p* = 0.880]. The heterogeneity, as assessed by the *I*^2^ statistic, was 95.3% (*p* < 0.0001).

## 4. Discussion

It is now widely accepted that Treg cells play a key role in the maintenance of immune tolerance and homeostasis [3, 45]. However, the role of Tregs in peripheral immune tolerance in patients with AS has not been fully elucidated in previous studies [7, 21, 31]. During the process, the markers used in the identification of Tregs are inconsistent by flow cytometry in previous studies; therefore, the proportion of peripheral Treg of AS patients has always been reported controversially [5, 24, 29, 46]. Our overall meta-analysis found no significant difference in Treg proportions between patients and healthy blood donors, although significant between-study heterogeneity was evident. We considered that the primary reasons for such unexpected results were due to inconsistent definitions of Tregs based on diverse markers used; thus, we subanalyzed the Treg data by the markers used for Treg identification, including CD25, FOXP3, and CD127.

Currently, researches on Tregs mainly focus on CD4^+^Tregs. Expression of CD25 (*α* chain of family IL-2R) correlates positively with Treg functionality [47]. The Treg-suppressive capacity is restricted to the CD4^+^ T cells that express the highest levels of CD25 [48]. We found out that AS patients had a higher proportion of Tregs termed “single CD25-positive” than had healthy blood donors. However, when Tregs were defined as “CD4^+^CD25^high^” or “CD4^+^CD25^bright^,” no significant differences were found between AS patients and healthy blood donors. And other activated CD4^+^ T cells also express CD25 [45, 48], indicating that use of the surface marker CD25 alone is inadequate. In 2008, Han G. et al. [49] found out that CD25^high^ cells that included a large proportion of FOXP3^−^ cells could not be classified as Tregs. The expressions of the transcription factor FOXP3 or other markers are considered more specific for the identification of Tregs than CD25 [50].

FOXP3 is a pivotal regulator of Treg fictional gene expression, being required for both Treg generation and survival [51]. The mutations of the FOXP3 gene disturb the function of Tregs, therefore resulting in the development of various autoimmune diseases [52]. Decreased FOXP3 expression causes an immune disease by subverting the suppressive function of Treg cells and converting Treg cells into effector cells [53]. However, when Tregs were defined as “FOXP3-positive” cells, the proportions of such cells did not differ between AS patients and healthy blood donors because the definitions of Tregs were complicated by the addition of CD25 status, giving “CD25-negative and FOXP3-positive” and “CD25 and FOXP3 double positive.” We also found that AS patients had a higher proportion of Tregs termed “CD4^+^CD25^low/−^FOXP3^+^” than had healthy blood donors. This phenomenon may be explained by the findings that the CD4^+^CD25^low/−^FOPX3^+^ cells were dysfunctional Tregs [54, 55] and may even be previously activated conventional T cells [56].

However, the detection of FOXP3 requires cell permeabilization, thereby preventing isolation of viable Tregs. Subsequently, the extracellular marker CD127 was established for the identification of Tregs [57–59]. Some scholars believe that CD4^+^CD25^+^CD127^low/−^ is the best surface marker of natural Tregs and alive Tregs, which not only can avoid interference of other activated T cells, but can also be used to conduct preliminary functional studies [19]. We found that the ratio of “CD127-negative” in peripheral blood of patients with AS was significantly lower than that of the control group, further suggesting that CD127 combined with other markers could indeed be used to label Tregs.

CD8^+^Tregs are similar to CD4^+^Treg and also have immunomodulatory effects. However, due to the lack of specific surface markers, few studies have been conducted on CD8^+^Treg [60, 61]. In 2015, Churland G. et al. [61] have found that the proportion of CD8^+^Tregs in the peripheral blood of healthy people is less than one-tenth of that of CD4^+^Tregs, which makes the study of CD8^+^Tregs more difficult. In this study, we found that only one study [21] reported the expression of CD8^+^Treg in peripheral blood of patients with AS, and a comprehensive analysis showed that there was a higher proportion of CD8^+^Treg in AS group than in the healthy control group. The specific marker, expression, and function of CD8^+^Treg need further study.

Some studies have used other markers to indicate different subsets of Tregs [24, 46, 62]. Human FOXP3^+^ cells have been subdivided into three functionally and phenotypically distinct subsets [63]: naïve Tregs (FOXP3^+^CD45RA^+^), short-lived and highly suppressive activated Tregs (FOXP3^high^CD45RA^−^), and non-Tregs (FOXP3^low^ CD45RA^−^). Although human naturally occurring Tregs may express either CD45RA or CD45RO, the majority of natural Tregs in adults are CD45RO^+^, which increases significantly with age [64, 65]. Ye L. et al. [62] found that in AS patients, the frequencies of effector Tregs (CD4^+^FOXP3^high^CD45RO^+^) and naïve Tregs (CD4^+^FOXP3^low^CD45RO^−^) were decreased. T-bet is an immune cell-specific member of the T-box family of transcription factors, which is required for the functional fitness of pTregs (also known as induced or adaptive Tregs [66–68]). Only Bidad K. [46] observed that FOXP3^+^CD4^+^ROR*γ*t^−^Tbet^−^ Tregs in AS patients were significantly lower than in healthy blood donors. A specialized subset of Tregs that are characterized by a high expression level of CXC chemokine receptor 5 (CXCR5), T follicular regulatory (Tfr) cells are important for the control of humoral immune responses [69, 70]. To date, it is still challenging to value the real status of above Treg subsets in patients with AS.

Further, the controversial status of Tregs in PB of AS patients might also be related to the different disease status, such as different treatment, disease activity, or markers of inflammation. It appears that the effects of corticosteroids (CS) on Treg numbers in patients with autoimmune diseases are disease-specific [71]. Treg cell numbers increased in CS-treated patients with SLE [72, 73] but decreased in CS-treated patients with psoriasis [74] and was not clearly defined in multiple sclerosis patients [75, 76]. Some studies found that disease-modifying antirheumatic drugs (DMARDs) can normalize the distribution of Tregs in RA patients [77–79]. Long-term anti-TNF therapy may increase Tregs in AS and other autoimmune diseases [41, 62, 80]. However, studies about CS and DMARDs on peripheral blood Tregs in AS patients are still lacking. In addition to drugs, disease activity also affects the proportion of peripheral blood Tregs [81, 82]. But our subgroup analysis found no difference in the proportion of Tregs in patients with active compared with stable AS. However, this conclusion needs to be confirmed by more studies on the proportion of Tregs and the activity of AS. One study also observed that the highest correlation coefficient was between CD4^+^CD25^+^FoxP3^+^Tregs and CRP or ESR [31]. But the true relationship between Tregs and inflammatory markers needs further studies.

Our meta-analysis had several limitations. Firstly, severity of the disease and clinical subtypes in AS patients were not consistent across studies. Moreover, although we did a subgroup analysis of disease activity, the results are questionable due to the small number of studies included. Second, we did not consider disease duration or treatment, as both the drugs used and disease staging were inconsistent; however, these factors might affect the proportion of Tregs in PB. Thirdly, there were differences in experimental methods between studies. A flow cytometric expert must run through all experiments: Some of the flow cytometric assays in the papers used here might even be disqualified. Meanwhile, the definition of Tregs in some studies also included CD127^low^ or CD25^high^ rather than completely the same definition makers. Moreover, Tregs are usually evaluated in PB, in which tissue Treg status may fluctuate.

## 5. Conclusion

Our study suggests that the reported variations of Treg status among AS patients are due to using inconsistent definitions or markers for Tregs. We found the best definition of Tregs as CD4^+^CD25^+^FOXP3^+^ or CD4^+^CD25^high/+^CD127^low/−^. Further studies are needed to validate our results in independent cohorts of patients with larger sample sizes using the above definitions of Tregs as accurate and standard definition of Tregs. Our findings lend support to the idea that the Treg status of AS patients is important.

## Figures and Tables

**Table 1:** Characteristics of the individual studies included in the meta-analysis.

Author (ref.)	Publish year	Country	EL^a^	*Q* ^b^	Case numbers		Tregs' definition	% of Tregs among CD4^+^ T cells [mean (or median) ± SD]		
					AS	HC		AS	HD	*p*
Duojia Cao et al. [14]	2004	Sweden	4	6	10	29	CD25^bright^CD4^+^	1.31 ± 0.68	1.23 ± 0.64	ns
Jau-Ling Suen et al. [17]	2008	Taiwan, China	4	6	23	36	CD4^+^CD25^high^FOXP3^+^	0.97 ± 0.33	0.86 ± 0.39	ns
Éric Toussirot et al. [32]	2009	France	4	6	32	15	CD4^+^CD25^+^FOXP3^+^	8.2 ± 0.61	7.94 ± 1.04	ns
Frauke Forger et al. [29]	2009	Swiss	4	7	15	18	CD4^+^CD25^high^	2.22 ± 1.47	2.12 ± 1.42	<0.01
Francesco Ciccia et al. [22]	2010	Italy	4	8	18	15	CD4^+^CD25^high^	1.08 ± 0.4	0.25 ± 0.12	<0.05
Christian Dejaco et al. [23]	2010	Austria	4	5	22	17	CD4^+^CD25^high^	13.54 ± 16.55	3.08 ± 2.48	ns
Heiner Appel et al. [33]	2011	Germany	4	6	19	20	CD4^+^FOXP3^+^	5.55 ± 2.54	5.18 ± 1.99	ns
Ming-Han Chen et al. [30]	2011	Taiwan, China	4	7	23	25	CD4^+^CD25^high+^	2.18 ± 0.11	2.16 ± 0.1	ns
Yanfeng Wu et al. [5]	2011	China	4	8	51	49	CD4^+^CD25^+^FOXP3^+^	1.23 ± 0.13	2.56 ± 0.16	<0.001
S-S Zhao et al. [6]	2011	China	4	8	14	18	CD4^+^CD25^high^CD127^low/−^	0.57 ± 0.29	1.65 ± 0.75	<0.001
Katayoon Bidad et al. [46]	2012	Iran	4	7	18	18	CD4^+^FOXP3^+^ ROR*γ*t^−^Tbet^−^	9.7 ± 1.2	16.1 ± 3	0.048
Leonardo Limon-Camacho et al. [11]	2012	Mexico	4	5	39	25	CD4^+^FOXP3^+^	7.3 ± 1.3	5.3 ± 1.7	0.01
Yong Gao et al. [34]	2012	China	4	8	40	37	CD4^+^CD25^+^FOXP3^+^	3.77 ± 0.81	4.69 ± 1.23	<0.05
Li Xueyi et al. [7]	2013	China	4	6	222	68	CD4^+^CD25^+^FOXP3^+^	2.14 ± 0.44	4.99 ± 0.49	<0.001
Lingying Ye et al. [62]	2013	China	4	6	21	22	CD4^+^CD45RO^+^FOXP3^high^	0.48 ± 0.07	0.73 ± 0.07	<0.05
Wei Ji et al. [39]	2014	China	4	7	20	20	CD4^+^CD25^+^CD127^low^	40.1 ± 17.5	58.6 ± 10.2	<0.05
Zhang Xin et al. [8]	2014	China	4	5	10	10	CD4^+^CD25^+^FOXP3^+^	2.72 ± 0.26	5.17 ± 0.31	<0.001
Hsien-Tzung Liao et al. [31]	2015	Taiwan, China	4	8	69	30	CD4^+^CD25^+^FOXP3^+^	1.73 ± 1.08	1.51 ± 0.48	<0.001
Yuxing Shan et al. [24]	2015	China	4	6	20	10	CD4^+^FOXP3^+^CXCR5^+^	5.57 ± 1.28	3.08 ± 0.59	<0.0001
Chenggong Wang et al. [35]	2015	China	4	6	45	20	CD4^+^CD25^+^FOXP3^+^	1.81 ± 0.81	1.23 ± 0.52	ns
Elliott TJ Dunn et al. [36]	2016	New Zealand	4	7	6	10	CD4^+^FOXP3^+^CD25^high^	1.43 ± 0.37	0.43 ± 0.15	ns
Huifang Guo et al. [9]	2016	China	4	8	39	17	CD4^+^CD25^high^FOXP3^+^	5.62 ± 0.4	5.89 ± 0.2	ns
Zhongliang Duan et al. [40]	2017	China	4	7	21	16	CD4^+^CD25^+^CD127^low^	2.7 ± 0.8	3.47 ± 0.83	0.03
Zofia Gula et al. [37]	2017	Poland	4	7	48	23	CD4^+^FOXP3^+^	28.83 ± 11.71	34.39 ± 20.65	ns
Dan Xu et al. [25]	2017	China	4	7	17	93	CD4^+^CD25^+^FOXP3^+^	22.58 ± 12.8	35.57 ± 6.48	<0.01
Mingfei Wang et al. [10]	2018	China	4	7	26	26	CD4^+^CD25^+^ FOXP3^+^CD127^−^	6.32 ± 1.5	5.44 ± 1.02	<0.05
Mohammad Javad Fattahietal [38].	2018	Iran	4	7	30	15	CD4^+^CD25^+^FOXP3^+^	2.7 ± 0.23	3.3 ± 0.47	0.45
Renfang Han et al. [21]	2018	China	4	6	40	40	CD8^+^CD122^+^	10.72 ± 6.32	1.21 ± 0.82	<0.05
Sonja Dulic et al. [41]	2018	Hungary	4	8	22	10	CD4^+^CD25^+^CD127^–^	5.708 ± 2.05	5.715 ± 0.79	ns

AS: ankylosing spondylitis; HD: healthy donors. ^a^Evidence level (EL) of each study was based on Oxford Centre for Evidence-Based Medicine 2011. ^b^Quality (*Q*) of each study was based on the Newcastle-Ottawa Quality.

**Table 2:** Subgroup analysis based on different definitions of Tregs in PB of patients with AS.

Definition of Tregs	Number of studies	Test of association			Test of heterogeneity		Egger's test	
		SMD	95% CI	*p* value	*I* ^2^	*p* value	*t*	*p* value
Single CD25-positive	6	0.736	(0.138, 1.334)	0.016	80.7%	<0.001	0.72	0.513
CD4^+^CD25^+^	1	0.846	(0.401, 1.291)	<0.001	–	–	–	–
CD4^+^CD25^high^	4	0.892	(−0.078, 1.862)	0.071	87%	<0.001	2.74	0.112
CD4^+^CD25^bright^	1	0.123	(−0.596, 0.842)	0.737	–	–	–	–
Associated with FOXP3-positive	18	−1.004	(−1.966, −0.042)	0.041	97.9%	<0.001	0.97	0.795
CD4^+^FOXP3^+^	3	0.383	(−0.663, 1.429)	0.473	90.4%	<0.001	−11.62	0.143
CD4^+^CD25^+^FOXP3^+^	9	−2.856	(−4.645, −1.066)	0.002	98.6%	<0.001	6.06	0.42
CD4^+^CD25^high^FOXP3^+^	3	0.868	(−0.756, 2.492)	0.295	92.6%	<0.001	2.91	0.862
CD4^+^CD25^low/−^FOPX3^+^	3	0.683	(0.161, 1.206)	0.01	68.4%	0.042	9.58	0.783
Associated with CD127-negative	4	−1.003	(−1.713, −0.294)	0.006	73.1%	0.011	−0.37	0.747
CD4^+^CD25^high^CD127^low/−^	1	−1.812	(−2.648, −0.977)	<0.001	–	–	–	–
CD4^+^CD25^+^CD127^low^	2	−1.12	(−1.605, −0.635)	<0.001	0.0%	0.486	–	–
CD4^+^CD25^+^CD127^−^	1	−0.004	(−0.751, 0.744)	0.992	–	–	–	–

PB: peripheral blood; AS: ankylosing spondylitis; SMD: standard mean difference; CI: confidence interval; *I*^2^: *I*-squared index. Magnitude of Cohen's *d* effect size (SMD): 0.2–0.5, small effect; 0.5–0.8, medium effect; and ≥0.8, large effect.

## Supplementary Materials

Supplementary Materials Supplementary Table 1: background of AS patients in each study. Supplementary Figure 1: forest plot of the overall meta-analysis of regulatory T cell (Treg) proportions in peripheral blood (PB), identified as single CD25-positive, between ankylosing spondylitis (AS) patients and healthy blood donors (HD). Supplementary Figure 2: forest plot of the overall meta-analysis of regulatory T cell (Treg) proportions in peripheral blood (PB), identified with FOXP3-positive, between ankylosing spondylitis (AS) patients and healthy blood donors (HD). Supplementary Figure 3: forest plot of the overall meta-analysis of regulatory T cell (Treg) proportions in peripheral blood (PB), identified with CD127-negative, between ankylosing spondylitis (AS) patients and healthy blood donors (HD).
