# Supplementary material for: The Proportion of Regulatory T Cells in Patients with Ankylosing Spondylitis: A Meta-Analysis
Source: J Immunol Res. 2019 Oct 23;2019:1058738. doi: 10.1155/2019/1058738 (PMC6854227; doi:10.1155/2019/1058738)

Supplementary Materials

Supplementary Table 1: Background of AS patients in each study.

Supplementary Figure 1: Forest plot of the overall meta-analysis of regulatory T cell (Treg) proportions in peripheral blood (PB), identified as single CD25-positive, between ankylosing spondylitis (AS) patients and healthy blood donors (HD).

Supplementary Figure 2: Forest plot of the overall meta−analysis of regulatory T cell (Treg) proportions in peripheral blood (PB), identified with FOXP3−positive, between ankylosing spondylitis (AS) patients and healthy blood donors (HD).

Supplementary Figure 3: Forest plot of the overall meta-analysis of regulatory T cell (Treg) proportions in peripheral blood (PB), identified with CD127-negative, between ankylosing spondylitis (AS) patients and healthy blood donors (HD).

Supplementary Table 1: Background of AS patients in each study.

Author (ref.)	Age, year	Female, %	Disease duration, year	ESR, mm/h	CRP, mg/l	BASDAI	Medication
Duojia Cao et al.[14]	36.6±12.4	60	NA	NA	NA	NA	NA
Jau−Ling Suen et al.[17]	43±12	17.4	NA	NA	NA	NA	NA
Éric Toussirot et al.[32]	42.9±1.1	26.7	9.2±0.6	28.2±2.1	25.1±3.2	35.9±2.2	NA
Frauke Forger et al.[29]	32.47±5.12	100	8.13±5.19	NA	77.1±50.4	3.03±2.14	NSAIDS<br>GC<br>Sulfasalazine<br>Cyclosporine
Francesco Ciccia et al.[22]	19-48	38.9	0.33±0.17	NA	NA	6.8±2.5	NSAIDS
Christian Dejaco et al.[23]	40.9±12.7	59.1	NA	23.1±23.6	11±20.4	NA	NA
Heiner Appel et al.[33]	40.9±13.8	NA	11.3±9.1	32.7±21.7	20±39	3.44±2.16	NSAIDS<br>MTX<br>TNF-α blocker
Ming−Han Chen et al.[30]	36.7±3	21.7	8.04±1.8	24.57±4.39	18.4±4.1	NA	TNF-α blocker
Yanfeng Wu et al.[5]	30.2±8.3	15.7	NA	NA	NA	≥4	NA
S−S Zhao et al.[6]	26.4±6.1	7.1	1.6±0.4	15.2±5.4	63±28	5.1±1.1	no drug
Katayoon Bidad et al.[46]	34±2	27.8	10±1	NA	NA	3.73±2.23	NSAIDS<br>DMARDS

Leonardo Limon−Camacho et al.[11]	32±13	18	NA	NA	14±3.2	4.4±2.4	NA
Yong Gao et al.[34]	29.1±8.6	35	4.3±1.9	NA	NA	NA	NA
Li Xueyi et al.[7]	33.6±8	31.1	NA	41±15.3	8.7±3.9	6.8±2.6	NSAIDS<br>DMARDS<br>TNF-α blocker
Lingying Ye et al.[62]	NA	NA	NA	NA	NA	NA	NA
Wei Ji et al.[39]	NA	NA	NA	31.18±15.63	16.3±11.28	51.94±11.57	NSAIDS<br>DMARDS
Zhang Xin et al.[8]	NA	NA	NA	NA	NA	NA	NA
Hsien−Tzung Liao et al.[31]	39.6±12.7	17.3	13.3±10.9	27.7±26	22±1.67	4.08±1.89	NA
Yuxing Shan et al.[24]	24.8±7.2	20	NA	57.3±23.2	40.2±26.5	5.2±0.7	NA
Chenggong Wang et al.[35]	51.8±7.5	48.9	3.04±2.5	NA	NA	NA	NA
Elliott TJ Dunn et al.[36]	51.3±15.7	0	7.8±6.8,2-21	NA	10±6.9	4.9±2	NSAIDS<br>MTX<br>Leflunomide<br>TNF-α blocker
Huifang Guo et al.[9]	28.2±1.04	19.7	NA	NA	NA	NA	NA
Zhongliang Duan et al.[40]	37±9.8	33.3	NA	NA	NA	>4	NA
Zofia Gula et al.[37]	33.9±6.8		6.67±3.01	24.78±6.7	7.95±3.22	2.93±1.14	NA
Dan Xu et al.[25]	NA	NA	NA	NA	NA	>4	NSAIDS<br>DMARDS

							Biological agents
Mingfei Wang et al.[10]	33.5±8.4	25	NA	39.8±14	26.3±12.7	5.9±1.5	NA
Mohammad Javad Fattahietal.[38]	31.4±9.1	26.7	11.8±8.6	NA	NA	≥4	GC<br>NSAIDS<br>MTX
Renfang Han et al.[21]	32.53±9.76	22.5	3.0±2.11	18.0±8.99	6.63±0.63	1.97±1.31	NSAIDS<br>DMARDS<br>Biological agents
Sonja Dulic et al.[41]	45.22±10.04	NA	1.72±0.87	23.64±18.84	23.45±26.06	1.19±4.62	TNF-α blocker

AS = Ankylosing spondylitis ; CRP = C-reactive protein; ESR = erythrocyte sedimentation rate; BASDAI = Bath Ankylosing Spondylitis Disease Activity Index; NSAIDS = Non-Steroidal Antiinflammatory Drugs; DMARDs = disease modified anti-rheumatic-drugs; MTX = methotrexate; NA = not applicable; GS = Glucocorticoids

Supplementary Figure 1

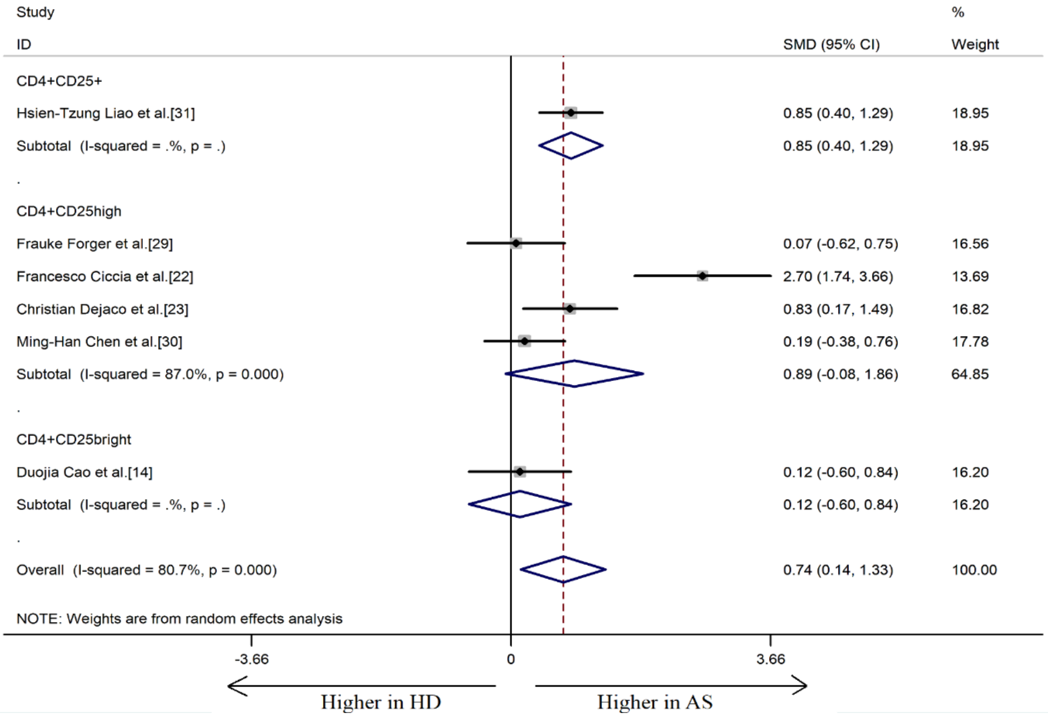

Supplementary Figure 2

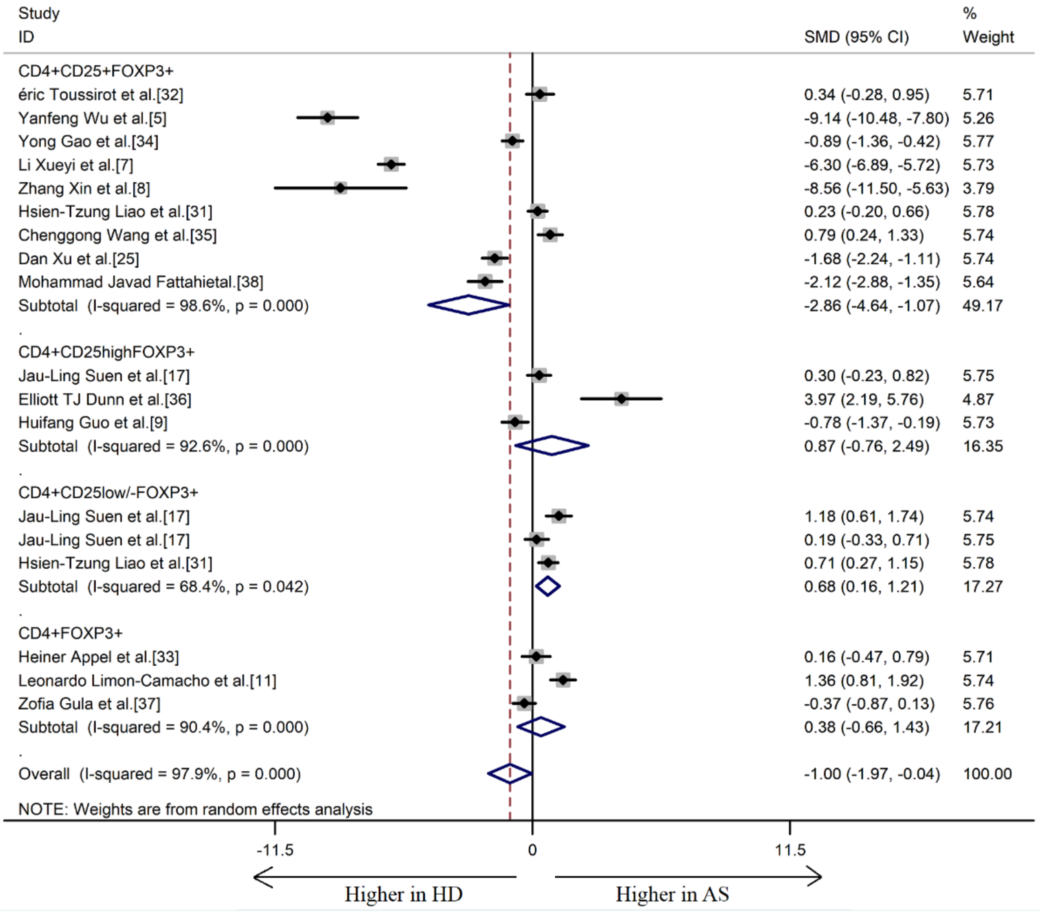

Supplementary Figure 3

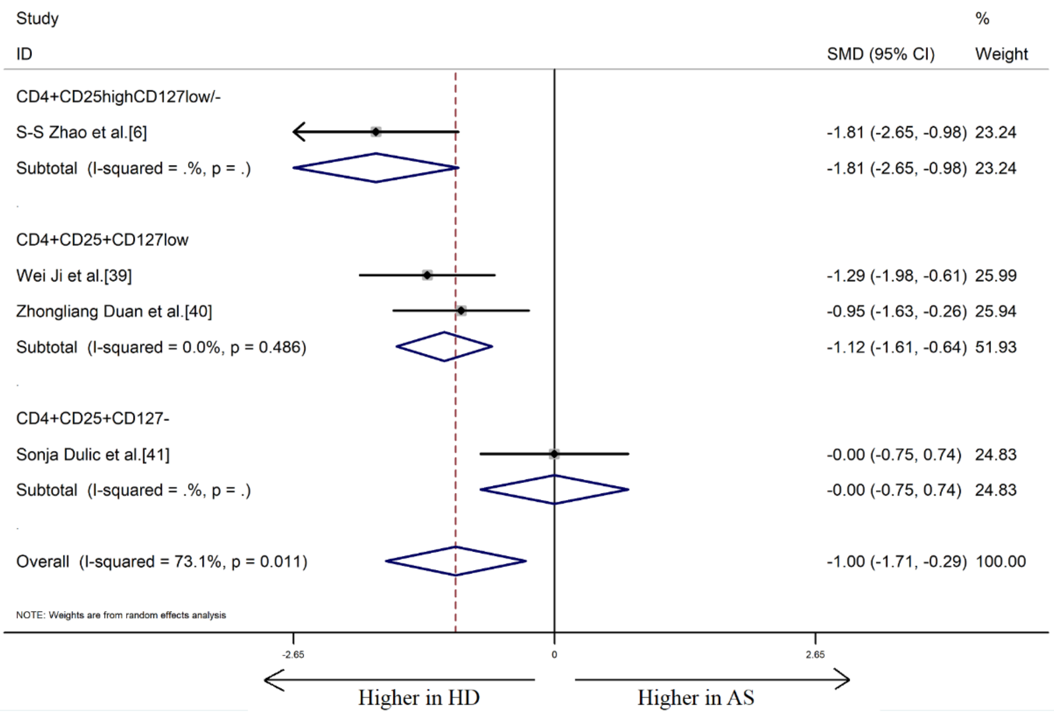

Supplement: Supplementary Materials — Supplementary Table 1: background of AS patients in each study. Supplementary Figure 1: forest plot of the overall meta-analysis of regulatory T cell (Treg) proportions in peripheral blood (PB), identified as single CD25-positive, between ankylosing spondylitis (AS) patients and healthy blood donors (HD). Supplementary Figure 2: forest plot of the overall meta-analysis of regulatory T cell (Treg) proportions in peripheral blood (PB), identified with FOXP3-positive, between ankylosing spondylitis (AS) patients and healthy blood donors (HD). Supplementary Figure 3: forest plot of the overall meta-analysis of regulatory T cell (Treg) proportions in peripheral blood (PB), identified with CD127-negative, between ankylosing spondylitis (AS) patients and healthy blood donors (HD). [file 1058738.f1.pdf]
